# The trouble with illness. How illness and disability affect relationships

**DOI:** 10.1080/17571472.2017.1397589

**Published:** 2017-11-07

**Authors:** Paul Thomas

**Affiliations:** LJPC Editor

If you are in a relationship with someone who is sick and you can’t understand why you are unhappy, you will find this book helpful. The book shows that it is common when illness strikes to develop a habit of negative thoughts and harmful actions. Case studies in the book show that many become angry and confused from incapacity, pain, rejection and loss, and these change the way we think. The author uses quotes and anecdotes from people she has counselled over 35 years to show how anxiety caused by illness can warp our subconscious minds and lead us to behave towards others in damaging ways. We project our inner ‘phantasies’ (p.44) – ideas, beliefs, stories, mental models, that we use to make sense of the world – onto others, behaving in rejecting, un-listening, unkind and unfair ways towards them. This behaviour is common in sick people and in their families and carers.

As well as helping people to understand the damaging effects that illness can have on relationships, the author wants to contribute to ‘understanding how people feel and react in the context of illness or damage to the body…. not simply to understand the world but to change it’ (p.13). This aim to facilitate positive change is particularly interesting to me as a GP since I often see patients who want to find positive things in their otherwise negative illness experience. I have often seen illness stimulate positive, revitalisation of relationships, so I know it is possible. But I have also seen it precipitate negative distancing, sometimes even frank abuse, both from the sick to the carer and the carer to the sick. I want to know how to chart a positive course. I found this aim to be less well served by the book, perhaps because the book focuses on those who sought counselling rather than those who did not, and perhaps because it focuses on the feelings of individuals rather than actions people can take together to build happy, loving relationships.

The book does suggest some actions that people can take to enhance relationships during illness - for example, quality communication and doing creative things together. Humour can help to say distressing things in more tolerable ways (p.97). Intimacy is especially important, including cuddles, hugs and touch as well as sexual intimacy to affirm that ‘the body as well as the mind is wanted, loved, cared for and functioning in a good way’ (p.135). The book reminds us to be honest but gentle when explaining to children what is happening, and avoid projecting our anxieties onto them (p.247).

I found particularly interesting the description of two common ways that people respond to anxiety that cause them to dissociate themselves from others and become inward-looking – depressive and paranoid-schizoid ‘positions’. The depressive position passively weakens relationships through withdrawal that sucks the joy out of relationships. The paranoid-schizoid (p-s) position actively destroys relationships - underlying panic and ‘massive fears’ cause them to be selfish, lacking consideration for others and losing reasoned thought (p.75). They make simplistic judgements that others are ‘good’ or ‘bad’, and imagine that others threaten them. Underlying this malady often lies huge, ‘life-threatening’ self-blame that is covered up by blaming others, often those most close to them, coupled with an inability to apologise or forgive.

Even without illness, it seems to me that large numbers of adults (and pre-adults) exhibit the accusing features of the p-s position and the withdrawn features of depression. As a consequence they are poor at developing loving relationships. If they are doing this because of inner anxieties, the implications for care and prevention are far-reaching. They need to acknowledge that they are causing the harm from their personal phantasies and get help to change them. Everyone in society needs to know how to manage anxiety in healthy ways.

I think that this book will be useful to those who are struggling to make sense of specific things that are affecting them. To improve those things, you may need to pick from different parts of the book the practical actions you can take to use illness as an opportunity to build strong, loving relationships. You may need to change the way you think and act.

